# Supplementary material for: Ciliary neurotrophic factor-mediated neuroprotection involves enhanced glycolysis and anabolism in degenerating mouse retinas
Source: Nat Commun. 2022 Nov 17;13:7037. doi: 10.1038/s41467-022-34443-x (PMC9672129; doi:10.1038/s41467-022-34443-x)
Supplement: Supplementary file 1 — Supplementary Information [file 41467_2022_34443_MOESM1_ESM.pdf]

Supplementary Figure 1
Metabolomics heatmap of WT, rds, and rds retinas treated with LV-IG or LV- CNTF

Rds retinas were treated with LV-IG or LV-CNTF from P25 to P36. Retinal tissues were incubated with 5mM glucose for 60 minutes prior to metabolomics analysis. Independent retina samples: N=6 for WT; N=4 for rds; N=6 for rds treated with LV-IG or LV-CNTF.

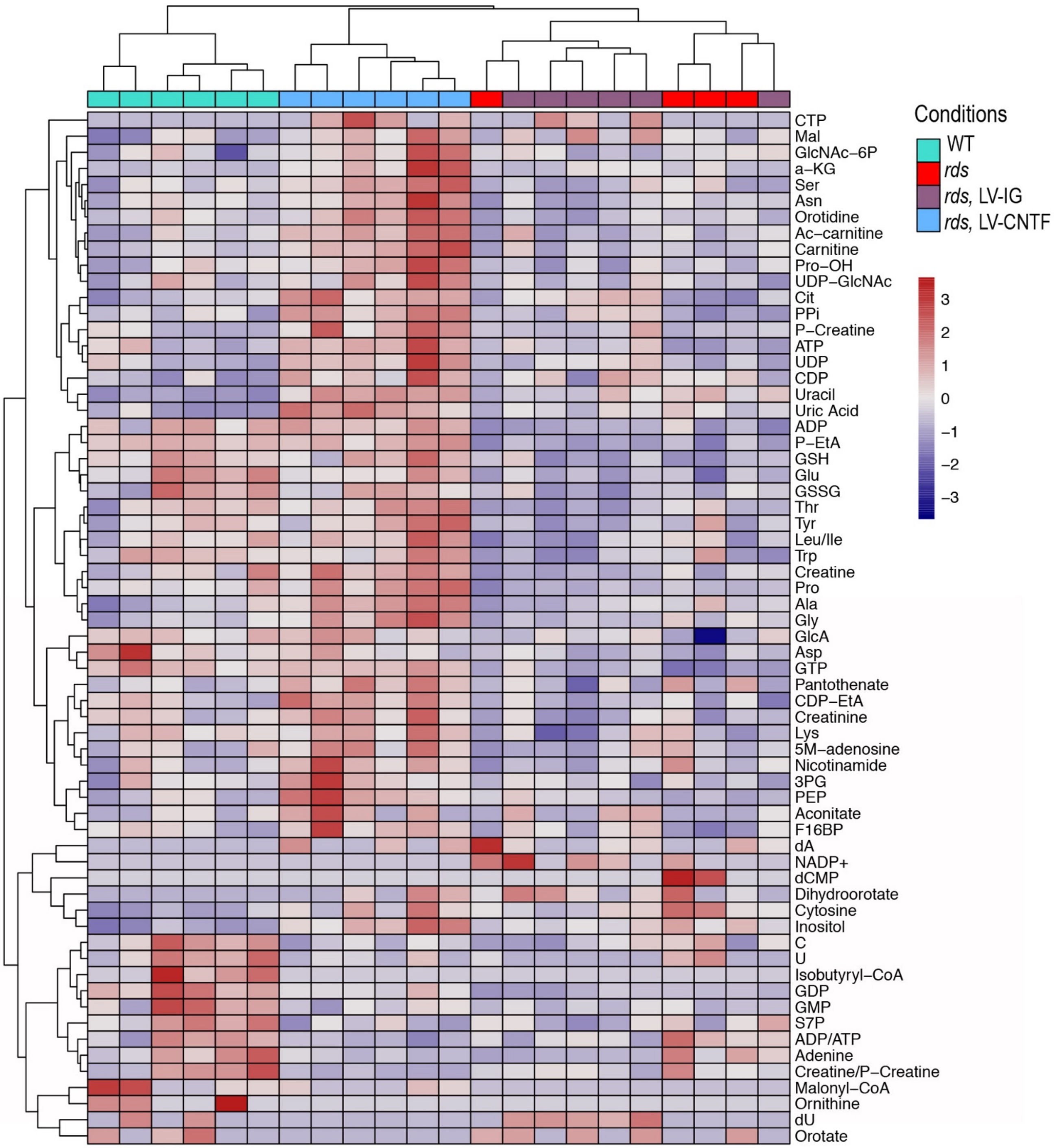

Supplementary Figure 2
Bar graphs of glycolysis, TCA cycle, currency, and fatty acid metabolites

Rds retinas were treated with LV-IG or LV-CNTF from P25 to P36. Retinal tissues were incubated with 5mM glucose for 60 minutes prior to metabolomics analysis. Independent retina samples: N=6 for WT; N=4 for rds; N=6 for rds treated with LV-IG or LV-CNTF. For **a-d**, data are presented as mean + SEM. One-way ANOVA was used to detected statistical significance in each metabolite group. * P < 0.05; ** P < 0.01; *** P< 0.001.

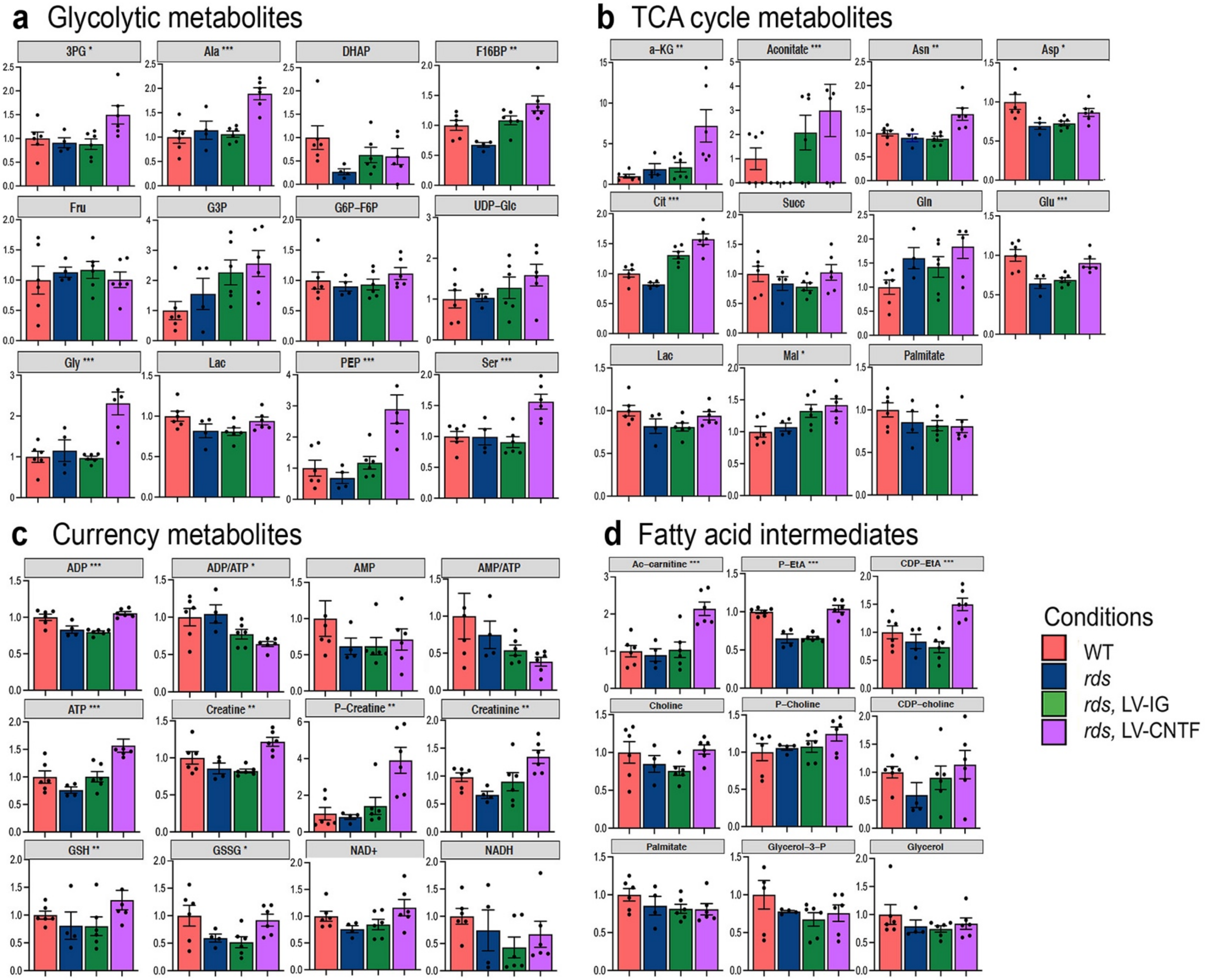

Supplementary Figure 3
Bar graphs and heatmap of amino acids with glucose as a nutrient

Rds retinas were treated with LV-IG or LV-CNTF from P25 to P36. Retinal tissues were incubated with 5mM glucose for 60 minutes prior to metabolomics analysis. Independent retina samples: N=6 for WT; N=4 for rds; N=6 for rds treated with LV-IG or LV-CNTF. **a.** Bar graphs of retinal contents of amino acids. Data are presented as mean ± SEM. One-way ANOVA was used to detected statistical significance in each metabolite group. * P < 0.05; ** P < 0.01; *** P< 0.001. **b.** Heatmap of relative amounts of amino acids.

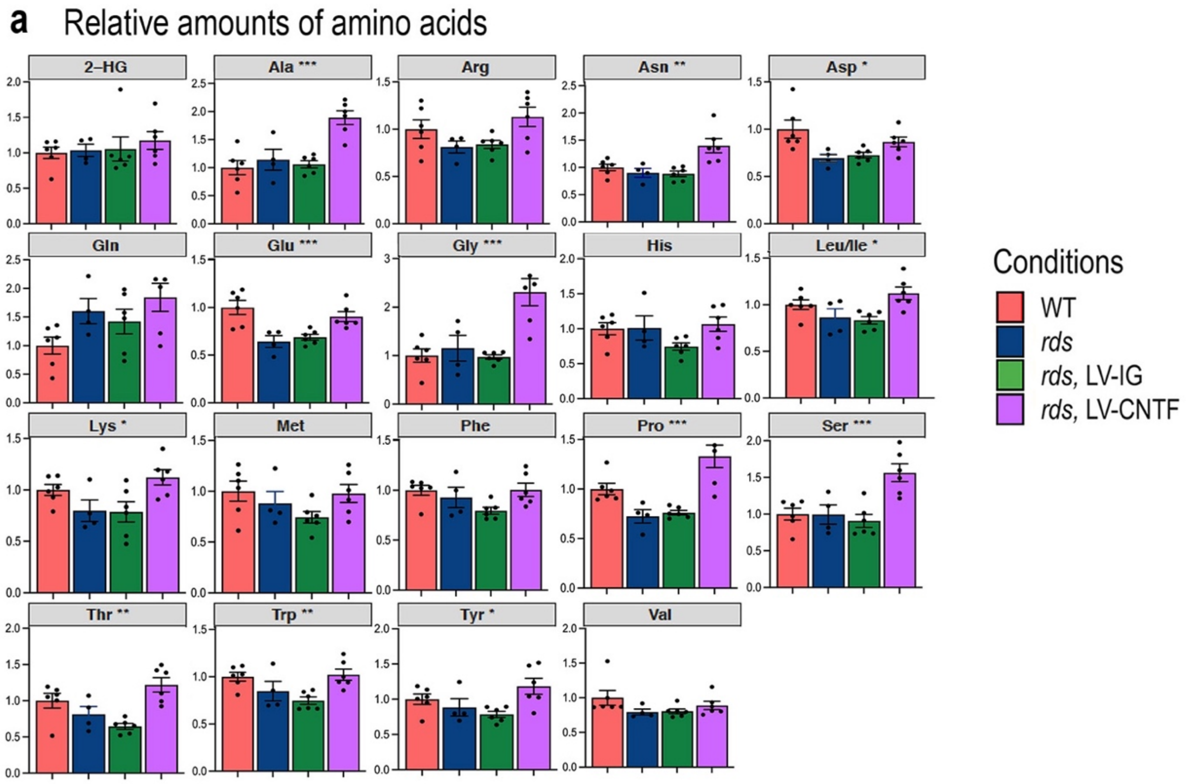

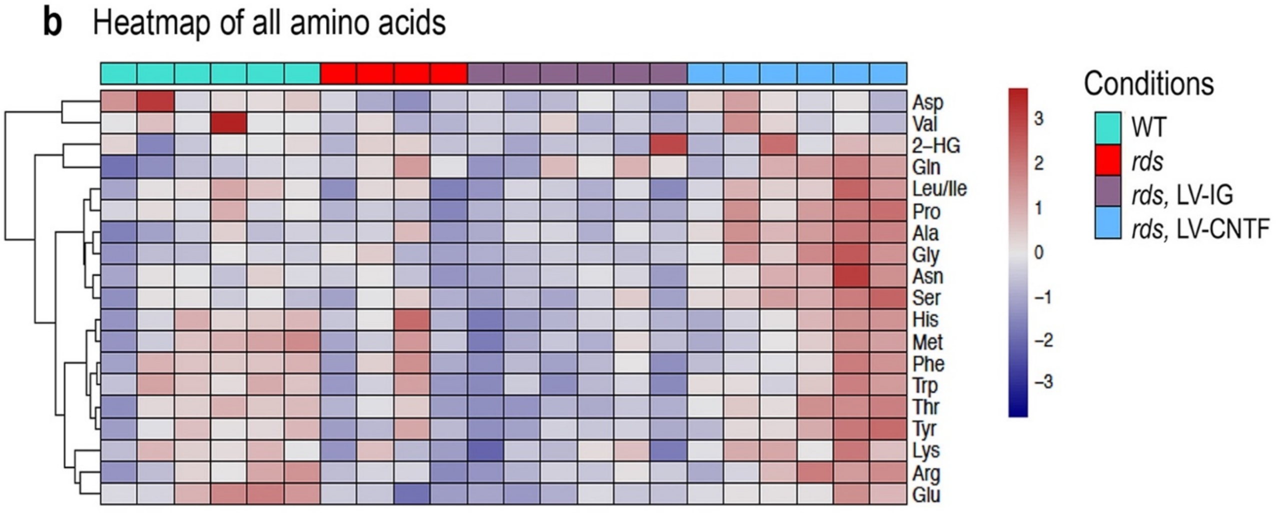

Supplementary Figure 4
Bar graphs of nucleotides and derivatives with glucose as a nutrient

Rds retinas were treated with LV-IG or LV-CNTF from P25 to P36. Retinal tissues were incubated with 5mM glucose for 60 minutes prior to metabolomics analysis. Independent retina samples: N=6 for WT; N=4 for rds; N=6 for rds treated with LV-IG or LV-CNTF. Data are presented as mean $\pm$ SEM. One-way ANOVA was used to detected statistical significance in each metabolite group. * $P < 0.05$; ** $P < 0.01$; *** $P < 0.001$.

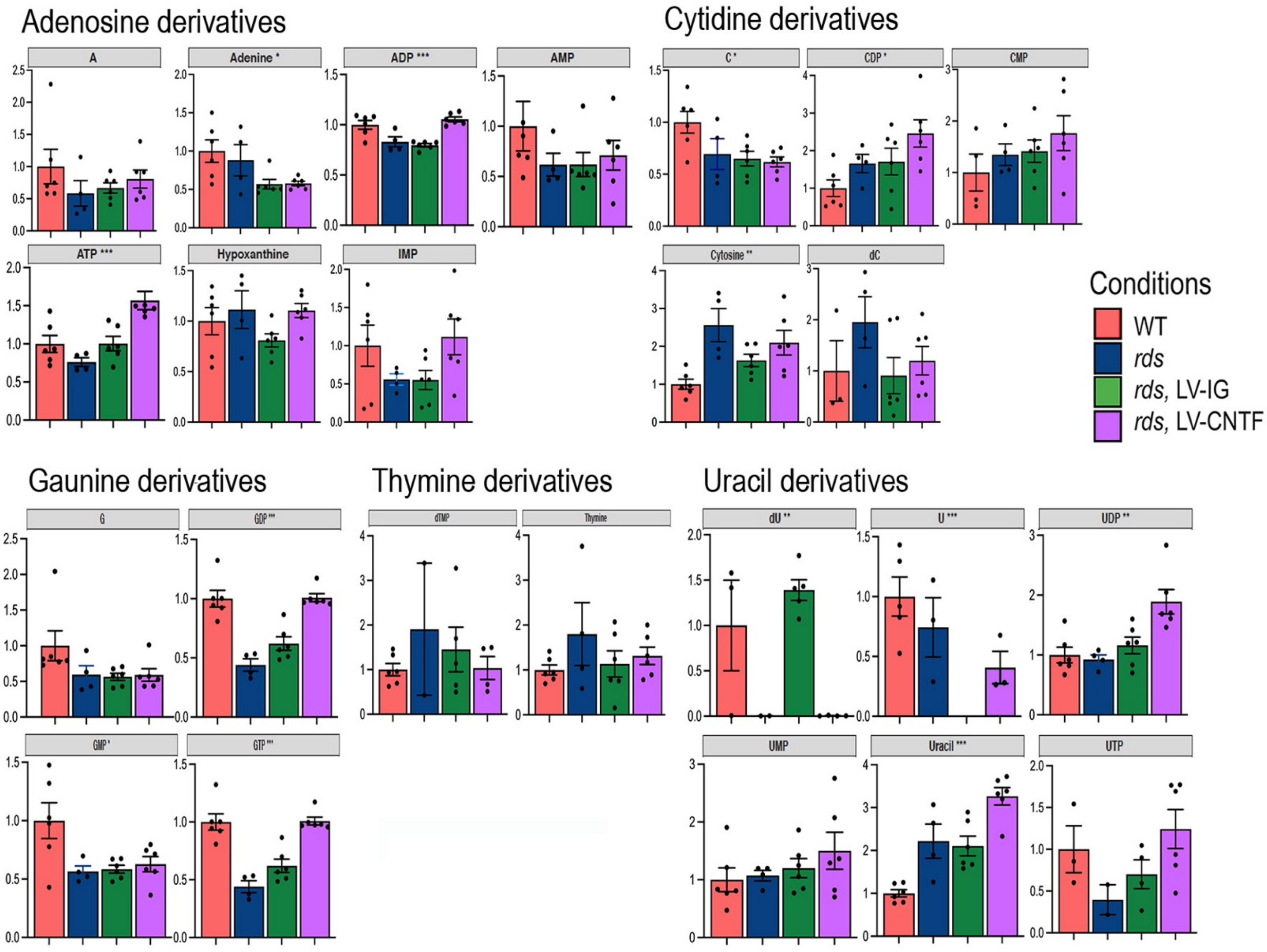

Supplementary Figure 5
Metabolomics heatmap of WT, rds, and rds retinas treated with LV-IG or LV-CNTF and Na^+Azide

Rds retinas were treated with LV-IG or LV-CNTF from P27 to P53. Retinal tissues were incubated with 5mM glucose for 60 minutes in the presence or absence of Na^+Azide. Independent samples: N=3 for WT+azide; N=4 for WT-azide, rds +/-azide, rds treated with LV-IG +/-azide; N=5 for rds treated with LV-CNTF +/-azide.

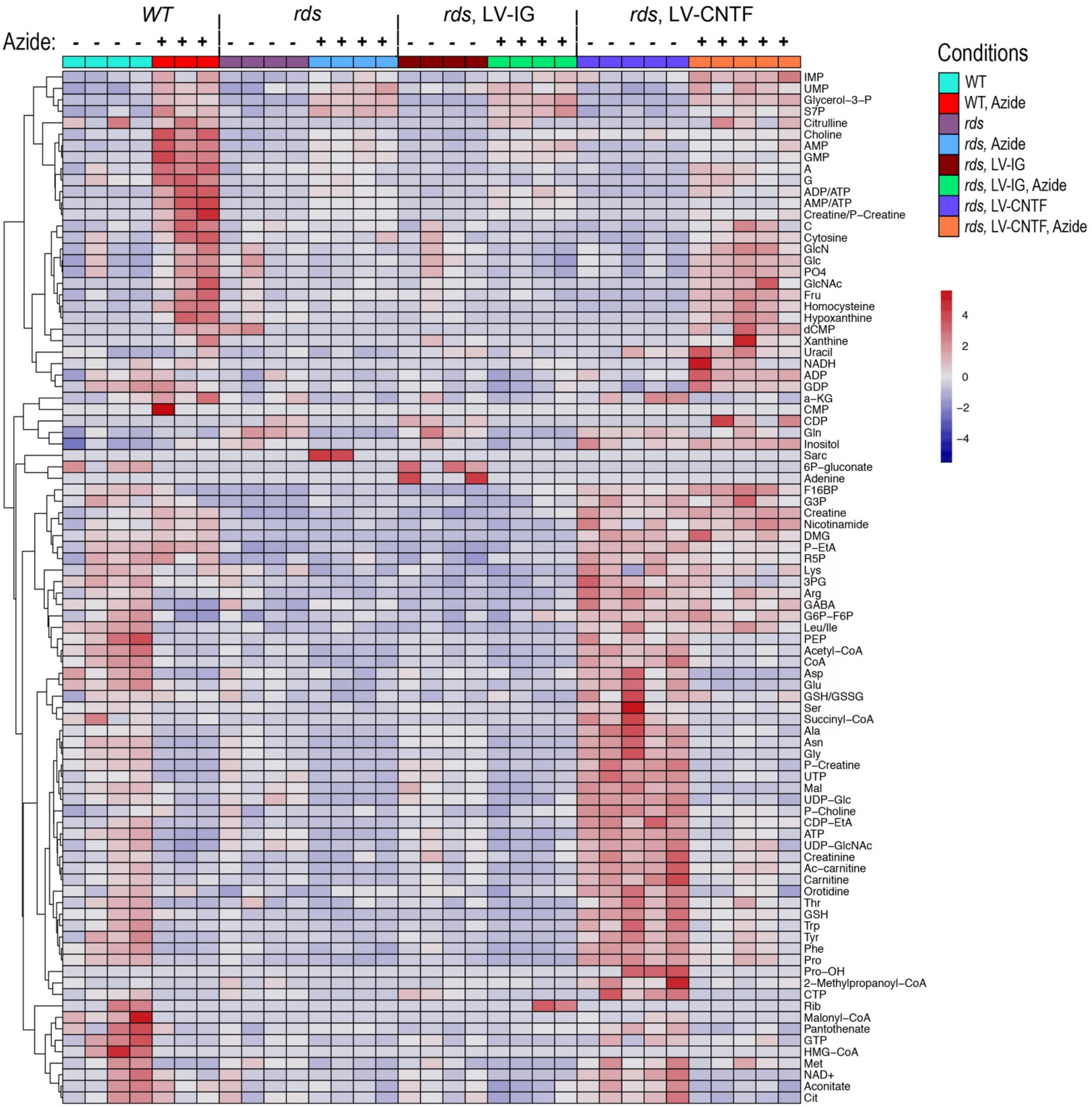

Supplementary Figure 6
Metabolomics heatmap of amino acids with glucose and Na^{+}Azide

Rds retinas were treated with LV-IG or LV-CNTF from P27 to P53. Retinal tissues were incubated with 5mM glucose for 60 minutes in the presence or absence of Na^{+}Azide. Independent samples: N=3 for WT+azide; N=4 for WT-azide, rds +/-azide, rds treated with LV-IG +/-azide; N=5 for rds treated with LV-CNTF +/-azide. Relative amounts of amino acids in retinal tissues are shown as a heatmap.

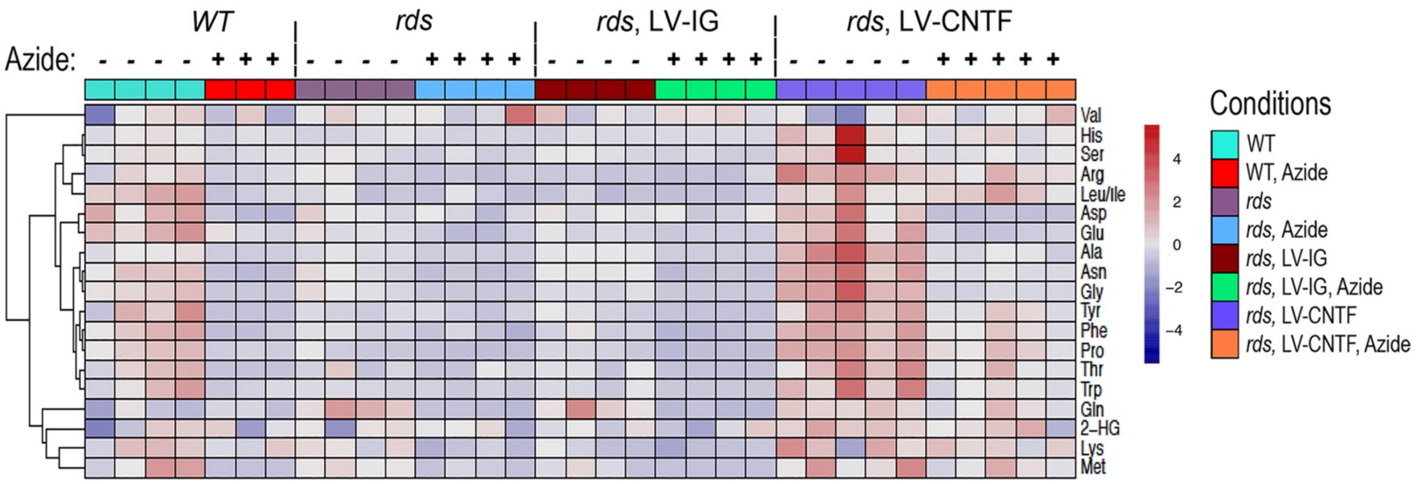

Supplementary Table. Summary of Reagents

PCR Primers for Genotyping

Gene	Forward	Reverse
rds (P216L)	CCT GGA GTT GCG CTG T	GTC TTTTTC ATG AAG CAC C
PhAM	CTTCCCTCGTGATCTGCAAC	CGCGACACTGTAATTTCATACTG
Rho iCre	TCA GTG CCT GGA GTT GCG CTG TGG	CTT AAA GGC CAG GGC CTG CTT GGC

Agilent Analyzer Reagents for Isolated Mitochondria Assay

Port	Compound	Injection Volume	Injected Solution Conc.	Final Conc.
A	ADP	16.5 µl	40 mM	4 mM
B	Oligomycin	20 µl	27.75 µM	3 µM
C	FCCP	20 µl	41 µM	4 µM
D	Antimycin A /Rotenone	20 µl	22.5 µM/22.5 µM	2 µM /2 µM

Agilent Analyzer Reagents for Isolated Mitochondria Complex IV Activity Assays

Port	Compound	Injection Volume	Injected Solution Conc.	Final Conc.
A	ADP	16.5 µl	40 mM	4 mM
B	Antimycin A /Rotenone	20 µl	27.75 µM	2 µM
C	TMPD/Ascorbate	20 µl	10 mM/9.5 mM	1 mM/950 µM
D	Azide	20 µl	460 mM	40 mM

Metabolomics Assay Inhibitors and Substrates

Reagent	Target	Concentration	Source	Catalog Number
GSK2837808A	Lactate Dehydrogenase (LDHA, LDHB)	10 µM/DMSO	AdipoGen	AG-CR1-3685
UK5099	Mitochondrial Pyruvate Carrier (MPC)	10 µM/DMSO	AdipoGen	AG-CR1-3691
Etomoxir	Carnitine Palmitoyltransferase-1 (CPT-1a)	40 µM/water	AdipoGen	AG-CR1-3688
Sodium Azide	Complex IV	20 mM/water	Sigma	S8032
D-Glucose		5mM	Sigma	G7021
Sodium Palmitate		5mM	Sigma	P9767

Antibodies used for Western blots

Antibody	Type	Dilution for Western	Source	Catalog Number
LDHa/c	rb mAb	1:1000 (wb)	Cell Signaling Inc.	3558
Phospho-LDHa (Try10)	rb Ab	1:1000 (wb)	Cell Signaling Inc.	8176
STAT3	rb mAb	1:1000 (wb)	Cell Signaling Inc.	12640
Phospho-STAT3 (Tyr705)	rb Ab	1:1000 (wb)	Cell Signaling Inc.	9171
γ-Tubulin (GTU-88)	mAb	1:2000 (wb)	Sigma	T6557
Licor (anti-rabbit)	dAb	1:20000 (wb)	LiCor	925-32211
Licor (anti-mouse)	dAb	1:20000 (wb)	LiCor	925-68070
