# Supplementary material for: Ciliary neurotrophic factor-mediated neuroprotection involves enhanced glycolysis and anabolism in degenerating mouse retinas
Source: Nat Commun. 2022 Nov 17;13:7037. doi: 10.1038/s41467-022-34443-x (PMC9672129; doi:10.1038/s41467-022-34443-x)
Supplement: Supplementary file 3 — Description of Additional Supplementary Files [file 41467_2022_34443_MOESM3_ESM.pdf]

Description of Additional Supplementary Files

File Name: Supplementary Video 1

Description: SIM video shows PhAM-labeled rod mitochondria located in the inner segments in WT retina at P96. The video includes regions of the outer nuclear layer and photoreceptor inner segments. Thickness of the optic section, 5μm. Scale bar, 3μm.

File Name: Supplementary Video 2

Description: SIM video shows PhAM-labeled rod mitochondria located in the synaptic terminals of WT retina at P96. The video includes the outer nuclear layer and the outer plexiform layer. Thickness of the optic section, 5μm. Scale bar, 3μm.

File Name: Supplementary Video 3

Description: SIM video shows PhAM-labeled rod mitochondria of rds mutant retina at P96. The video includes the entire outer nuclear layer and inner segments. Thickness of the optic section, 5μm. Scale bar, 3μm.

File Name: Supplementary Video 4

Description: SIM video shows PhAM-labeled rod mitochondria of rds retina treated with LV-IG from P28-P96. The video includes the entire outer nuclear layer and inner segments. Thickness of the optic section, 5μm. Scale bar, 3μm.

File Name: Supplementary Video 5

Description: SIM video shows PhAM-labeled rod mitochondria of rds retina treated with LV-CNTF from P28-P96. The video includes part of the outer nuclear layer and inner segments. Thickness of the optic section, 5μm. Scale bar, 3μm.
