# Supplementary figures and images for: Ciliary neurotrophic factor-mediated neuroprotection involves enhanced glycolysis and anabolism in degenerating mouse retinas
Source: Nat Commun. 2022 Nov 17;13:7037. doi: 10.1038/s41467-022-34443-x (PMC9672129; doi:10.1038/s41467-022-34443-x)

8-26-2021

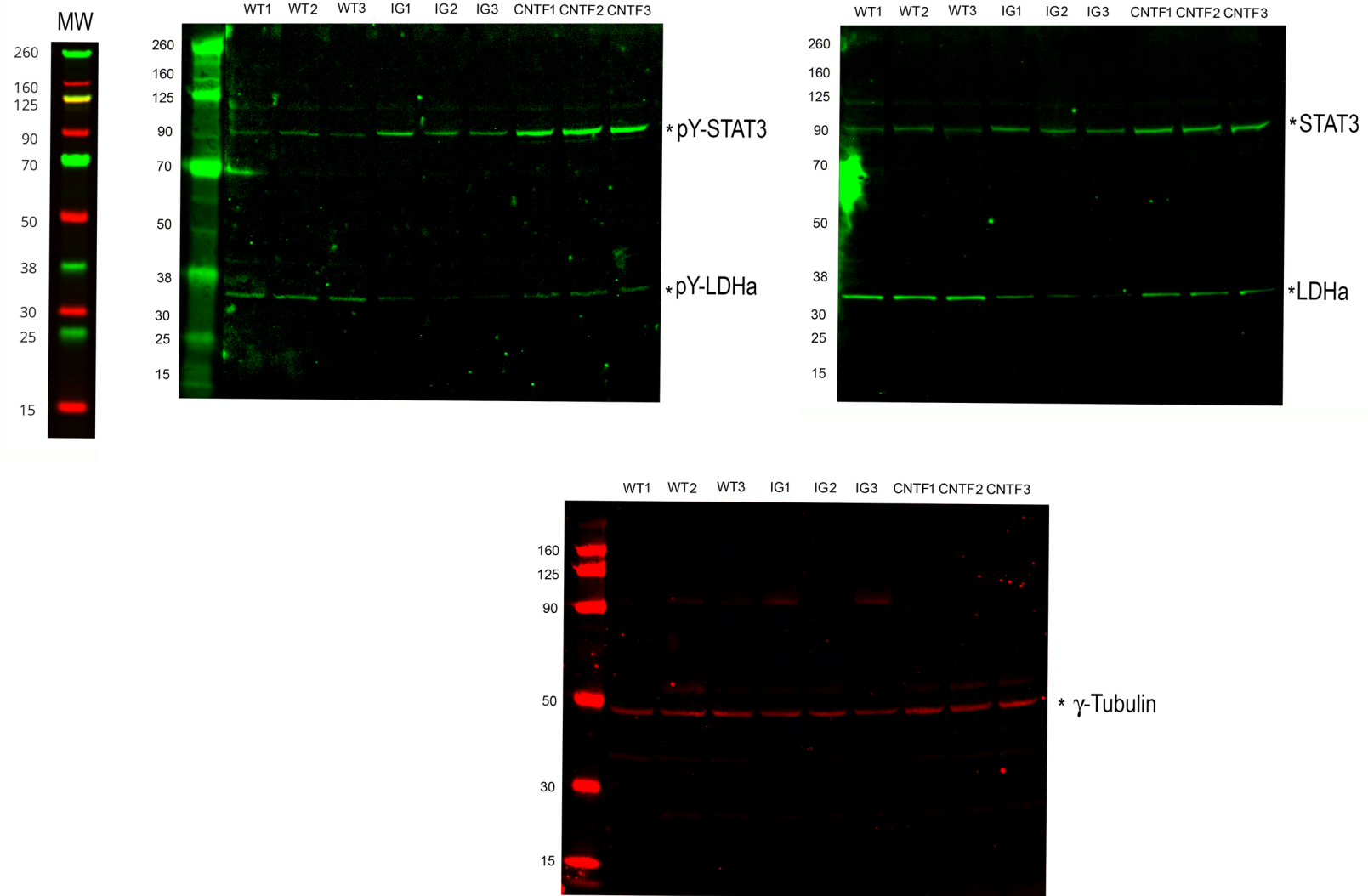

Supplement: Supplementary file 10 — Source Data [file 41467_2022_34443_MOESM10_ESM.zip › 337302_3_data_set_6991500_rjs4lc.pdf]
